# Density Functional Theory Applied to Excited State Intramolecular Proton Transfer in Imidazole-, Oxazole-, and Thiazole-Based Systems

**DOI:** 10.3390/molecules23051231

**Published:** 2018-05-21

**Authors:** Fabricio de Carvalho, Maurício D. Coutinho Neto, Fernando H. Bartoloni, Paula Homem-de-Mello

**Affiliations:** Centro de Ciências Naturais e Humanas, Universidade Federal do ABC, Santo André, São Paulo 5001, Brazil; fabricio.carvalho@ufabc.edu.br (F.d.C.); mauricio.neto@ufabc.edu.br (M.D.C.N.)

**Keywords:** ESIPT, DFT, hydrogen bonding, imidazole, oxazole, thiazole

## Abstract

Excited state intramolecular proton transfer (ESIPT) is a photoinduced process strongly associated to hydrogen bonding within a molecular framework. In this manuscript, we computed potential energy data using Time Dependent Density Functional Theory (TDDFT) for triphenyl-substituted heterocycles, which evidenced an energetically favorable proton transfer on the excited state (i.e., ESIPT) but not on the ground state. Moreover, we describe how changes on heterocyclic functionalities, based on imidazole, oxazole, and thiazole systems, affect the ESIPT process that converts an enolic species to a ketonic one through photon-induced proton transfer. Structural and photophysical data were obtained theoretically by means of density functional theory (DFT) calculations and contrasted for the three heterocyclics. Different functionals were used, but B3LYP was the one that adequately predicted absorption data. It was observed that the intramolecular hydrogen bond is strengthened in the excited state, supporting the occurrence of ESIPT. Finally, it was observed that, with the formation of the excited state, there is a decrease in electronic density at the oxygen atom that acts as proton donor, while there is a substantial increase in the corresponding density at the nitrogen atom that serves as proton acceptor, thus, indicating that proton transfer is indeed favored after photon absorption.

## 1. Introduction

The dynamics of excited state intramolecular proton transfer (ESIPT) has been investigated both experimentally and theoretically by different research groups [1,2,3,4,5,6,7,8,9,10,11,12,13,14,15,16], since the first observation of this phenomenon by Weller et al. in 1956 [17,18]. A large number of applications are based on ESIPT, such as laser dyes [19,20,21,22,23,24], solar energy concentrators [25], chemosensors [26,27], electroluminescent materials [28], photostabilizers [29,30], molecular probes [31], and metallic ion sensors [32,33,34,35].

ESIPT is a photoinduced process observed in organic compounds containing at least one potential proton donor group (e.g., –OH, –NH_2_, and NHSO_2_R) and an equivalent proton acceptor group (–N, –C=O, etc.), separated by no more than 2.0 Å [36]. The presence of the intramolecular hydrogen bond can directly influence the geometry of the molecules [37,38,39,40,41,42,43]. Moreover, intramolecular hydrogen bonding in the excited state would affect non-adiabatic processes that occur after photon absorption, such as internal conversion, intersystem crossing, intramolecular charge transfer, and so on [43,44].

**Scheme 1:**
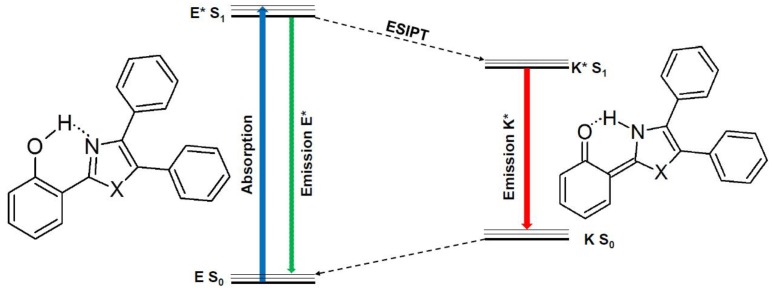
Excited state intramolecular proton transfer (ESIPT) mechanism within a triphenilimidazolyc framework. Adapted from reference [34].

ESIPT is more efficient as the acidity and/or basicity of donor and acceptor groups increase after photoexcitation [45,46,47]. Basically, molecules that undergo ESIPT have the thermodynamically favored enolic form in the ground state (S_0_, Scheme 1) [48], which is stabilized by the intramolecular hydrogen bond. After photoexcitation, the donor group acidity and the basicity of the acceptor core must increase simultaneously due to redistribution of charge density on the chromophore. This process allows the migration of the proton from the acid to the basic center, generating the phototautomer [49].

ESIPT is a very fast process occurring on a time scale of picoseconds [2] and even femtoseconds [50], depending on the experimental conditions. Particularly, the employed solvent [50,51] and certain substituents introduced in the molecule [50] can shift the ESIPT time scale, since the transition state barrier is affected by all these factors. Generally, ESIPT chromophores show dual emission, a normal emission from the enolic form, at lower wavelengths, and a second emission with large Stokes shift (above 8,000 cm^−1^) due to the tautomer formed after proton transfer [35] (Scheme 1). The excited enolic form (E*) can undergo a radiative decay and return to the enolic fundamental state (E), or it may undergo ESIPT to generate the excited ketone species (K*), which decays emitting at lower energy than the E* form; the large Stokes shift is due to substantial geometry reorganization [52].

**Figure 1:**
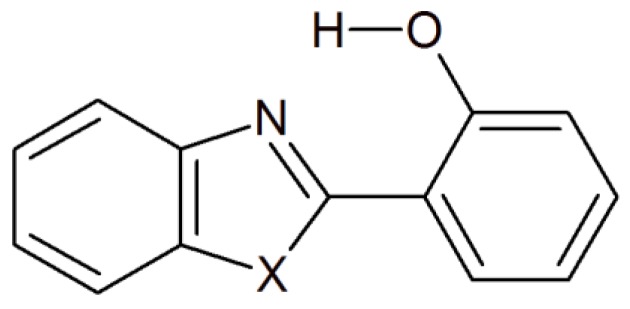
Enolic form for 2-(2’-hydroxyphenyl)benzoxazole (HBO) (X = O), 2-(2’-hydroxyphenyl)benzimidazole (HBI) (X = NH), and 2-(2’-hydroxyphenyl)benzothiazole (HBT) (X = S) [5].

Purkayastha et al. studied the rotamerization and the ESIPT process for 2-(2’-hydroxyphenyl)benzoxazole (HBO), 2-(2’-hydroxyphenyl)benzimidazole (HBI), and 2-(2’-hydroxyphenyl)benzothiazole (HBT, Figure 1) [5]. Three signals in the fluorescence spectra were observed for HBO and HBI, whereas HBT presented only two emission signals. The authors attributed this phenomenon to the existence of two rotamers for HBO and HBI, while there is only one for HBT. Intramolecular proton transfer for these three compounds is unfavorable in the ground state, both thermodynamically and kinetically, and these factors contribute to the occurrence of ESIPT in the lowest singlet and triplet states [5].

**Figure 2:**
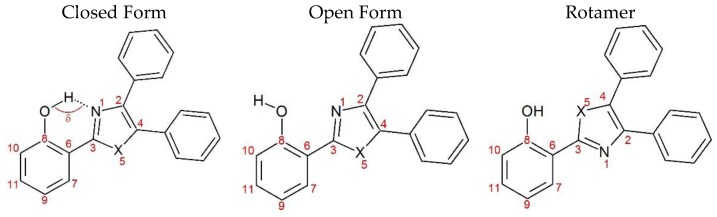
General structure for triphenylimidazole (N_E_, with X = NH), triphenyloxazole (O_E_, with X = O) and triphenylthiazole (S_E_, with X = S), and respective conformers.

Considering the various applications and importance of the ESIPT process for a wide class of molecules, this manuscript presents a theoretical study of the heterocyclic functionality effect in triphenylimidazole, triphenyloxazole, and triphenylthiazole systems (Figure 2). While experimental data only provides indirect information on the ESIPT mechanism and is often limited by the availability of synthesized compounds, computational methods are relevant tools to obtain structural and electronic properties, and also to give insight on these reactions mechanism. So, this work may shed light on the role of different heteroatoms on ESIPT processes.

## 2. Results and Discussion

The proton transfer mechanism and the role of the heteroatom for three proposed triphenyl-substituted compounds, 2-(4,5-diphenyl-1*H*-imidazol-2-yl)phenol (N_E_), 2-(4,5-diphenyl-1,3-oxazol-2-yl)phenol (O_E_), and 2-(4,5-diphenyl-1,3-thiazol-2-yl)phenol (S_E_), was investigated. Subscripts _E_ and _K_ are related to the enolic and ketonic species in the ground state; when these are accompanied by an * mark, they refer to the corresponding species in the excited state (i.e., _E*_ and _K*_). Therefore, N_E*_ refers to the enolic excited state of the triphenylimidazole system, while O_K_ to the ketonic ground state of the triphenyloxazole one, and so on.

Table S1 presents and compares the calculated data for the S_E_ species, with the data for HBT (Figure 1) from Purkayastha et al. [5] together with its corresponding crystallographic data [53]. It can be seen that the bond distances and angles of the heterocyclic functionality are very similar, although there are differences on the molecular framework on a larger scale for these triphenylthiazole and benzothiazole systems.

Table 1 shows the obtained values of free energy differences for the studied N_E_, O_E_, and S_E_ triphenyl-substituted derivatives. The closed form (Figure 2) of the enolic species was considered as the reference for all cases (Table 1), and it can be verified that the open structure for the three heterocyclic systems is always less stable. Also for all derivatives, the rotamer is more stable than the open form, but less stable than the closed enolic species, indicating that intramolecular hydrogen bonding stabilizes the molecular framework, as expected, because a six-membered system is formed [54]. It is worth noting that O_E_ rotamer is more stable than the other rotamers due the O-H∙∙∙O hydrogen bonding established, but still less stable than the closed form. N_E_ and S_E_ are less stable since no effective hydrogen bonding is expected (in N_E_, the nitrogen atom in position 5, Figure 2, is also connected to hydrogen). As mentioned before, the compounds studied by Purkayastha et al. [5] may present both O_E_ and N_E_ rotamers, with energy differences smaller than 0.6 kcal mol^−1^ associated to the closed forms, while for the compounds studied here the relative energies are about ten times greater. So, further computational and experimental studies should be performed to confirm the existence of rotamers for the triphenylic compounds.

**Figure 3:**
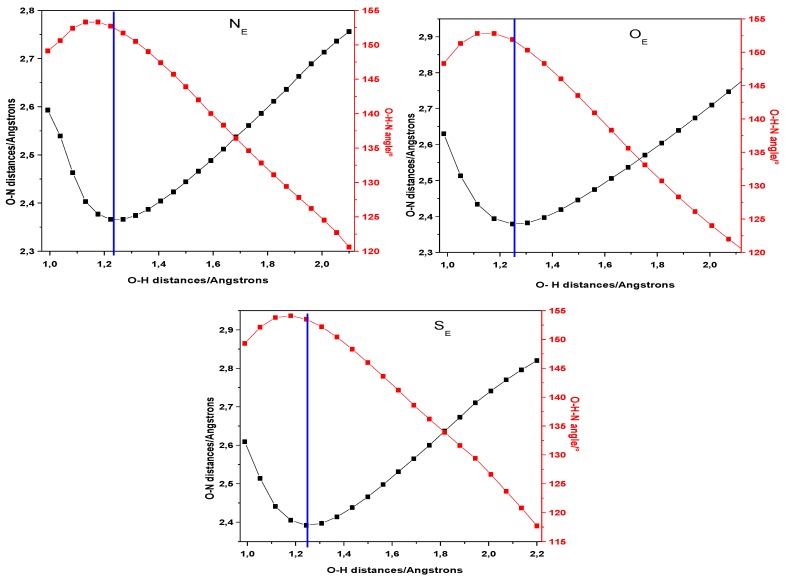
Variation of O–N distance (in black, left y-axis) and O–H∙∙∙N angle (in red, right y-axis) for N_E_, O_E_, and S_E_ with changes on the O–H distance. The blue line indicates in which O–H distance the proton is transferred.

The analysis of how the proton transfer coordinate advances is a crucial point for the construction of the potential energy curves in the ground and excited states. Initially, the geometries of the molecules in the enolic species, both in the ground state and in the excited state, were minimized without any geometric restriction. To obtain the ketone tautomer (K), it was necessary to constrain the N–H bond distance during geometry optimization. So, potential energy curves for the conversion from enolic to ketonic species, both in fundamental (S_0_) and in the first excited state (S_1_), were constructed based on a relaxed scan for the variation of the O–H bond, with a 0.06 Å step, and, for each step, vertical transitions were calculated with TD-DFT using the same basis set and functional as for the ground state calculation. For each step, we have evaluated the influence of the O–H bond distance over the O–H∙∙∙N angle and the distance between the oxygen and nitrogen atoms (O–N distance), for compounds N_E_, O_E_, and S_E_ (Figure 3). At low O–H bond lengths the decrease in the distance between the oxygen and nitrogen atoms occurs gradually and then reaches a minimum at 1.24 Å for N_E_, 1.26 Å for O_E_, and 1.25 Å for S_E_, where the proton is transferred. After proton transfer, the distance between the phenolic oxygen atom and the nitrogen of the heterocyclic functionality increases again. The opposite occurs for the variation of the O–H∙∙∙N with O–H bond length: before proton transfer the angle increases, while a decrease is observed for larger O–H distances (Figure 3).

**Figure 4:**
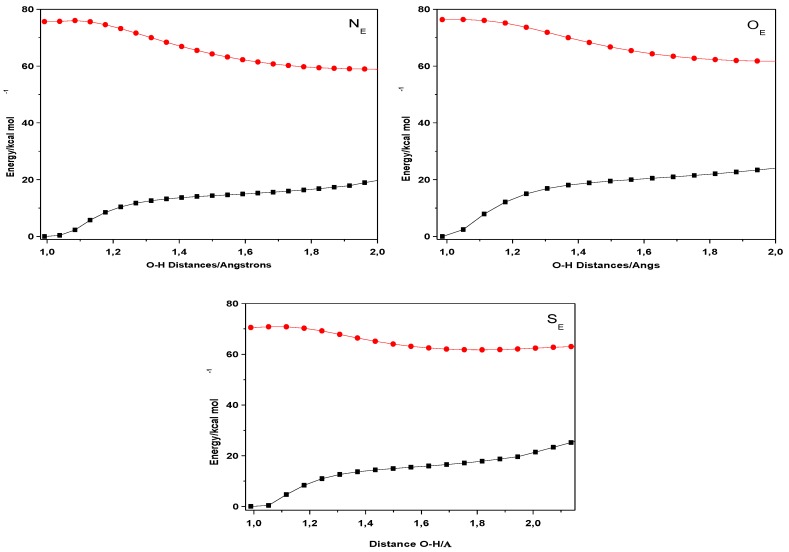
Calculated potential energy curves for the S_0_ (in black) and S_1_ (in red) states of N_E_, O_E_, and S_E_, respectively, along the proton transfer coordinate.

The potential energy curves obtained here for the three systems (Figure 4) indicate that in the ground state proton transfer is not allowed: at the distance of ca. 1.2 Å (the distance of proton transfer), the energy difference regarding the enolic minima increases to about 16 (N_E_), 22 (O_E_), and 17 (S_E_) kcal mol^−1^. However, in the excited state, there is an energy barrier for proton transfer of 0.97, 0.02, and 0.29 kcal mol^−1^ for N_E*_, O_E*_, and S_E*_, respectively. These processes could even be considered barrierless, if the average errors associated to the method employed are taken into account, meaning that the ESIPT process goes smoothly for all cases. Moreover, substitution of a nitrogen atom by oxygen or sulfur makes the proton transfer even more favored, as seen by the aforementioned energy barriers at the excited state.

Table 2 contains the main structural parameters for the compounds as the enolic species, both in the fundamental (E) and excited (E*) states (optimized structures for enolic species are in the Supporting Information, Figure S1). For the three systems, O–H bond length increases in the excited state, when compared to the ground state, about 0.017, 0.019, and 0.014 Å, for N_E_, O_E_, and S_E_, respectively. This increment in bond distance in the excited state indicates that there is a strengthening of intramolecular hydrogen bonding between the H and N atoms. As mentioned in the Introduction, donor (O–H) and acceptor (N) groups are separated by distances smaller than 2.0 Å, about 1.7 Å in the ground state. The strengthening of the hydrogen bond can be expressed also by the decrease of N∙∙∙H distance: about 0.058, 0.073, and 0.064 Å, for N_E_, O_E_, and S_E_, respectively, which facilitates proton transfer [44,55,56,57]. This phenomenon is especially relevant for the O_E_ compound, for which there is the greatest variation in the distance and, consequently, the smallest barrier for proton transfer in the excited state. The δ_O–H∙∙∙N_ angle increases 0.9° for N_E_, 1.9° for O_E_, and 1.8° for S_E_, emphasizing that hydrogen bonding is stronger in the excited state, mainly for O_E_ and S_E_.

Analyzing the C_8_–O bond distance (Table 2), for the three studied systems, there was a decrease in this bond length in the excited state, in comparison to the ground state, of 0.017, 0.018, and 0.012 Å, for N_E_, O_E_, and S_E_, respectively. This indicates an increment of double bond character in the excited state, which is a characteristic of compounds that undergo ESIPT [58,59,60]. The highest carbon-heteroatom bond distances were observed for the triphenylthiazole system, particularly, for C_3_–S_5_ and C_4_–S_5_ (Figure 2), in both ground and excited states. This increase in bond length was expected because the atomic radius of sulfur is higher than that of the other heteroatoms (i.e., N and O) and its electronegativity is smaller, leading to this increase in bond distance. Also, for this thiol derivative, a more expressive increment of the bond distances in the excited state, in comparison to the ground state, is overall observed (about 0.02 Å, Table 2 and Table S2). Contrarily, for the imidazolic system, there is no systematic increase or decrease in bond lengths when the excited state is generated from the fundamental one.

From the theoretically obtained IR vibrational spectra (Figures S2–S4), the vibrational stretching frequency of the O–H bond in the ground state appears in the following sequence: 3225 cm^–1^ for N_E_, 3350 cm^−1^ for O_E_, and 3290 cm^−1^ for S_E_, in agreement with O–H bond length and δ_O–H∙∙∙N_ angle increment order. In the excited state, this order in energy is maintained between functionalities, but the vibrational transitions appear at lower wavenumbers, with a decrease of 265 cm^−1^ for N_E_, 235 cm^−1^ for S_E_, and only 85 cm^−1^ for O_E_. So, for the three studied systems, there was a shift towards the bathochromic region (i.e., lower energy) of the O–H group stretching signal, thus indicating that the intramolecular hydrogen bonding in O–H∙∙∙N is strengthened, mainly for N_E_ and S_E_.

Table 3 presents the experimentally determined p*K*_aH_ values from the literature for 1*H*-imidazole [61], 1,3-oxazole and 1,3-thiazole [62], thus, for the heterocyclic functionality without phenyl substituents; while protonated oxazole is the more acidic species, the imidazolic heterocycle is more basic. We have, then, obtained the difference in free energies for protonated and deprotonated forms of these molecules (i.e., ΔΔG) by frequency calculations. Even though to obtain the p*K*_aH_ theoretically further studies would be necessary, from the present data it can be already seen (Table 3), that the trend in ΔΔG values correspond to the one in p*K*_aH_, with a more negative free energy associated to an increased basicity.

By the data in Table 4, containing atomic charges derived from the electrostatic potential (calculated with the ChelpG scheme), it can be verified that both the enolic and ketonic species of the studied compounds present the oxygen atom of the phenolic group with a more negative charge than the basic nitrogen atom of the heterocyclic functionality. This contributes for a higher stability of the enol tautomer at the ground state (N_E_, O_E_, and S_E_) when compared to the corresponding ketonic species.

To deepen the study of the heteroatom influence over the electronic distribution both on ground and excited states, electronic transitions regarding absorption of light were simulated (Figures S5–S7). Experimentally, the triphenylimidazole compounds present two absorption signals, the first at 322 nm, attributed mainly to a HOMO → LUMO transition, and the second at 294 nm, corresponding mainly to a HOMO → LUMO + 1 transition [34]. Jayabharathi et al. obtained experimental absorption spectra with a well-defined band for some benzimidazole derivatives, which have a phenolic hydroxyl group hydrogen bonded intramolecularly with the nitrogen atom [63]. However, Wang et al. have obtained the absorption spectrum of a compound derived from benzothiazole which has two absorption bands, therefore, it can be seen that the features of the absorption spectrum may change according to the structure of the compound [64]. On the other hand, only one signal dominates the experimental emission spectra for two N_E_ derivatives (see for example references [34,35]), associated to the K* tautomer deactivation. Emission of the E* species occurs at shorter wavelengths, and can be experimentally observed when ESIPT is prevented due to complexation with metallic species [34,35].

**Figure 5:**
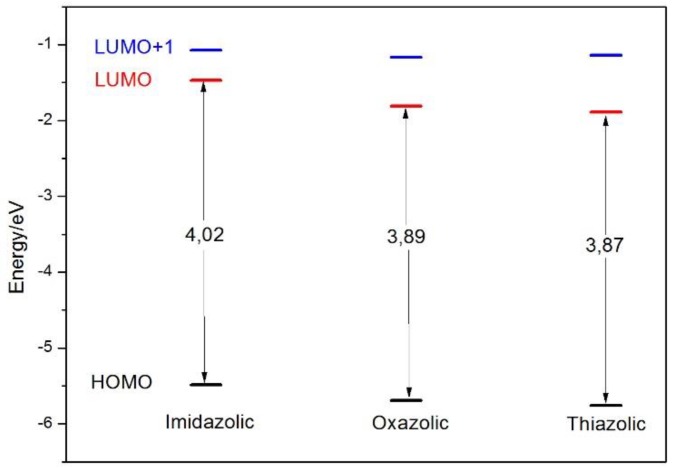
Representation of frontier orbital energy levels for N_E_, O_E_, and S_E_, respectively.

Particularly for the N_E_ triphenylimidazole derivative, an excellent agreement between experimental and calculated absorption data has been observed using the B3LYP functional [35]. B3LYP has been applied successfully [58,65,66,67,68,69,70] in similar systems, when compared to available experimental data, although this functional has been shown to present limitations due to its generality (see for example reference [71]). Therefore, we have evaluated the N_E_ absorption spectrum with different functionals (B3LYP, M06, wB97X-D3, and M06-2X, Table S2). Although there were no changes in molecular orbital contributions, B3LYP still was the best functional to adequately predict absorption data, followed by M06. For the compounds object of this study, the first transition (HOMO → LUMO character) occurs almost at the same region for O_E_ and N_E_, around 338 nm, and at a less energetic region for S_E_ (at 346 nm). However, when the same analysis is carried out with respect to the transition with HOMO → LUMO+1 character, this order is inverted: N_E_ exhibits a maximum absorption wavelength of 311 nm, followed by O_E_ at 305 nm and S_E_ at 303 nm (Table 5). Figure 5 indicates that these differences correspond to the decrease in the HOMO-LUMO energy gap in the following order N_E_ > O_E_ > S_E_, with the decrease in the LUMO energy level contributing more to changes in the energy gap. On the other hand, LUMO + 1 is less affected by changing the heteroatom, so the second transition is mainly affected by the smaller changes in the HOMO energy level.

**Figure 6:**
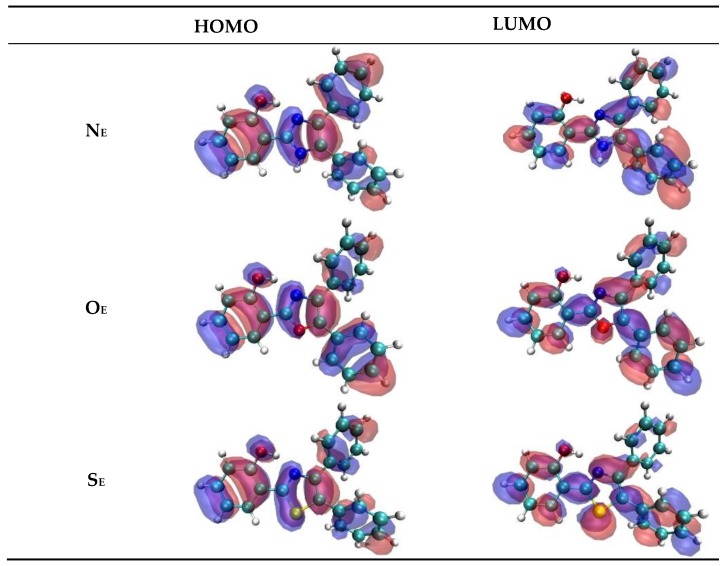
Molecular orbitals HOMO and LUMO for N_E_, O_E_, and S_E_, respectively.

Aiming to shed light on formation of excited state, we have evaluated HOMO and LUMO electron densities of the enolic species for N_E_, S_E_, and O_E_ (shown in Figure 6). It is verified that the HOMO of the three heterocyclic systems is distributed over the whole molecule. The HOMO → LUMO transition of the enolic species leads to a decrease in the electron density in the oxygen atom of the hydroxyl group. This trend must directly influence intramolecular hydrogen bonding, since, as previously seen, the interaction distance –H∙∙∙N= is decreased after photoexcitation to the S_1_ state, implying an increase in strength of the intramolecular hydrogen bond, corroborating the analysis of the infrared signals of S_0_ and S_1_ states [72]. However, S_E_ and O_E_ presented higher electron density on the hydroxyl oxygen atom than the electron density presented for N_E_, even though this density is smaller than in the respective HOMO orbitals.

It should be noted that the S_1_ state change in electron density in the hydroxyl group could directly influence intramolecular hydrogen bonding (Figure 6). The interaction between the electrons of the heterocyclic system and the non-bonding orbital of the OH group is the one mainly responsible for the transfer of proton from the oxygen atom to the nitrogen one [72]. Consequently, the excited state of the studied systems should favor the protonation of the nitrogen atom and, consequently, the deprotonation of the phenolic group (see Supporting Information for HOMO and LUMO of ketonic forms, Figures S8–S10).

Once the molecule reaches a stable conformation in the excited state, already as the ketonic species, emission of energy occurs, and a new barrier-free process happens, from ketone to enolic species in the ground state. Wang et al. studied the fluorescence of some 2-(2’-hydroxyphenyl)benzothiazole derivatives and showed that these compounds have dual fluorescence [64]. An emission that appears at low wavelengths and is related to the fluorescence of the enolic form, and another signal that appears at higher wavelengths and is associated to the emission of the ketonic form [64,73]. When using polar solvents, just a single emission was observed for imidazolic derivatives, related to the enolic species emission, however, when using non-polar solvents, the dual emission was observed [73].

The analysis of the emission data (Table 5) indicates that the N_E_ compound emits in a lower energy region, which shows that the gap between the excited and ground states is the most stabilized among the three studied systems, corresponding to an emission at 523 nm. For S_E_ and O_E_, the emission occurs at 490 and 452 nm, respectively, showing that the framework with the heterocyclic functionality containing the oxygen atom is the one with the highest energy difference between states. 

## 3. Materials and Methods

We have employed the Density Functional Theory (DFT) and the Time-Dependent Density Functional Theory (TD-DFT) to study the ESIPT process for N_E_, O_E_ and S_E_. These approaches have been widely used before to clarify fundamental aspects regarding the different structural and electronic states [13,59,65,73,74,75,76,77] as well as to study theoretically the intramolecular hydrogen bonding in the excited state for different compounds for whose ESIPT occurs [44,78,79,80,81,82,83].

As mentioned above, geometries of the enolic species (E and E*) were minimized without any geometric restriction, but to obtain the ketone tautomers (K and K*), it was necessary to constrain the N–H bond distance during geometry optimization. Vibrational frequencies of the enolic optimized structures were obtained to verify that these correspond to a minimum in the potential energy surface [84]. Besides that, frequency calculations were also used to obtain thermodynamic information for 1*H*-imidazole, 1,3-oxazole and 1,3-thiazole (protonated and deprotonated species), to calculate the free energy of protonation (based on the difference of the free energies of formation) aiming to give insight on experimental p*K*_aH_ values presented by the different heterocycles. TD-DFT calculations were performed to obtain the absorption spectra for both conformations using 40 states of excitation. Atomic charges were derived from electrostatic potential using ChelpG scheme. All calculations were performed with B3LYP [85,86,87] functional and the basis set 6-31G(2d,2p) [88] as implemented in Orca software [89], using an energy convergence criteria of 10^−8^ Hartree.

The potential energy curves for the proton transfer (conversion from enolic to ketonic species, both in S_0_ and in S_1_), were constructed at B3LYP/6-31G(2d,2p) level: for the ground state a relaxed scan for the variation of the O–H bond, with a 0.06 Å step was performed and, for each step, vertical transitions were calculated with TD-DFT. This technique was used before to construct potential energy curves in the excited state and has provided good results when compared to experimental data [58,65,66,67,68,69,70]. Emission wavelength in Table 5 was calculated based on the energy difference between the minimum at the excited state of ketonic form and the same geometry at the ground state.

## 4. Conclusions

DFT and TD-DFT calculations were applied to describe the ongoing ESIPT process in triphenylimidazole, triphenyloxazole, and triphenylthiazole systems. Intramolecular proton transfer in the ground state does not occur because there is an energy barrier greater than 10 kcal mol^–1^ separating the conversion of the enolic tautomer to the ketonic one. This barrier decreases significantly (<1 kcal mol^–1^) after excitation to the first excited state, enabling intramolecular proton transfer. Among three different possible conformations, the one corresponding to a closed enolic form is the most stable, since intramolecular hydrogen bonding leads to an overall stabilization of the molecule. For the three systems, O–H bond distance increases in the excited state following H∙∙∙N length decrease, demonstrating that in the excited state the intramolecular hydrogen bond is strengthened; this was corroborated by simulated vibrational data. The HOMO → LUMO transition energy gap increases when the heteroatom changes from S, to O and N in the heterocyclic system, while emission energy decreases for the N, S, O sequence. Electronic densities in the phenolic oxygen atom are high in the HOMO for all three systems as their enolic species, but these densities decrease after photoexcitation, marking the proton transfer. Therefore, the identity of the heterocyclic functionality is absolutely relevant on the context of intramolecular proton transfer within these frameworks. The findings reported on this study may contribute to the design of new relevant ESIPT-operating systems, particularly with respect to the identity of the heterocyclic system that acts as proton acceptor group.

## Figures and Tables

**Table 1:** Relative free energy, in kcal mol^−1^, for the N_E_, O_E_, and S_E_ in the ground state, in the closed, open, and rotamer forms.

	Closed Form (kcal mol^−1^)	Open Form (kcal mol ^−1^)	Rotamer (kcal mol^−1^)
**N_E_**	0	13.55	8.47
**O_E_**	0	12.55	5.77
**S_E_**	0	12.68	9.48

**Table 2:** Bond length (Å) and angle formed by O-H∙∙∙N (δ_O–H∙∙∙N_, ^o^) for enolic form of N_E_, S_E_, and O_E_ in ground state (S_0_ = _E_) and first excited singlet state (S_1_ = _E*_).

	N_E_	N_E*_	O_E_	O_E*_	S_E_	S_E*_
**O–H**	0.992	1.009	0.986	1.005	0.989	1.003
**H^…^N**	1.711	1.653	1.765	1.692	1.742	1.678
**X_5_–C_3_**	1.364	1.382	1.351	1.360	1.751	1.776
**X_5_–C_4_**	1.388	1.379	1.385	1.393	1.747	1.767
**δ_O–H∙∙∙N_**	148.8	149.7	147.0	148.9	147.9	149.7

Atom numbering from Figure 2.

**Table 3:** Theoretically obtained free energy differences between protonated and deprotonated forms of heterocyclic compounds and their respective experimental p*K*_aH_ values.

	1*H*-Imidazole	1,3-Oxazole	1,3-Thiazole
**ΔΔG (kcal mol^−1^)**	−230.61	−213.79	−219.25
**p*K*_aH_**	6.9 ^a^	0.8 ^b^	2.5 ^b^

Taken from references ^a^ [61] and ^b^ [62].

**Table 4:** ChelpG charges in the ground state for the enolic and ketonic species of the imidazolic, oxazolic, and thiazolic heterocyclic systems, regarding the atoms involved in intramolecular hydrogen bonding.

Atom	N_E_	N_K_	S_E_	S_K_	O_E_	O_K_
**N**	−0.4151	−0.2140	−0.4254	−0.2456	−0.3325	−0.1723
**O**	−0.5118	−0.6061	−0.4961	−0.5698	−0.4933	−0.5735
**H**	0.3309	0.3004	0.3280	0.3167	0.3177	0.2896

**Table 5:** Theoretical absorption wavelength (λ_abs_), Oscillator strength (*f*), contributions (CI), and theoretical emission wavelength (λ_emis_) of N_E_, O_E_, and S_E_, respectively.

Main Composition		λ_abs_ (nm)	*f*	CI (%)	λ_emis_ (nm)
**N_E_**	HOMO → LUMO HOMO → LUMO+1	337.5 310.6	0.3850 0.4288	94.2 86.6	523
**O_E_**	HOMO → LUMO HOMO → LUMO+1	339.1 304.9	0.6724 0.2266	94.2 78.4	452
**S_E_**	HOMO → LUMO HOMO → LUMO+1	346.5 302.8	0.5478 0.1188	93.6 86.3	490

## Supplementary Materials

The following are available online: Tables S1 and S2; Figures S1–S10.
