# Supplementary material for: Density Functional Theory Applied to Excited State Intramolecular Proton Transfer in Imidazole-, Oxazole-, and Thiazole-Based Systems
Source: Molecules. 2018 May 21;23(5):1231. doi: 10.3390/molecules23051231 (PMC6100175; doi:10.3390/molecules23051231)
Supplement: Supplementary file 1 [file molecules-23-01231-s001.pdf]

Supplementary Material

Density functional theory applied to excited state intramolecular proton transfer in imidazole-, oxazole- and thiazole-based systems

Fabricio de Carvalho [1], Maurício D. Coutinho Neto [1], Fernando H. Bartoloni [1,*] and Paula Homem-de-Mello [1,]**

[1] Centro de Ciências Naturais e Humanas, Universidade Federal do ABC, Santo André, São Paulo, Brazil.

Authors to whom correspondence should be addressed:
*fernando.bartoloni@ufabc.edu.br; Tel.: +55-11-4996-8375
**paula.mello@ufabc.edu.br; Tel.: +55-11-4996-0166

Table S1: Comparison between structural parameters of S_E and HBT from Purkayastha [5] and crystallographic data [78]. Bond length (Å) and angle formed (^{o}) in ground state

Structural parameters	Calculated	Purkayastha	Cryst. data
$C_8 – O$	1.342	1.366	1.305
$C_3 – N_1$	1.315	1.327	1.280
$C_2 – N_1$	1.379	1.400	1.404
$C_3 – S$	1.751	1.751	1.749
$C_4 – S$	1.747	1.687	1.757
$C_2 – N – C_3$	113.3	110.3	110.8
$C_3 – S – C_4$	90.1	91.0	88.6

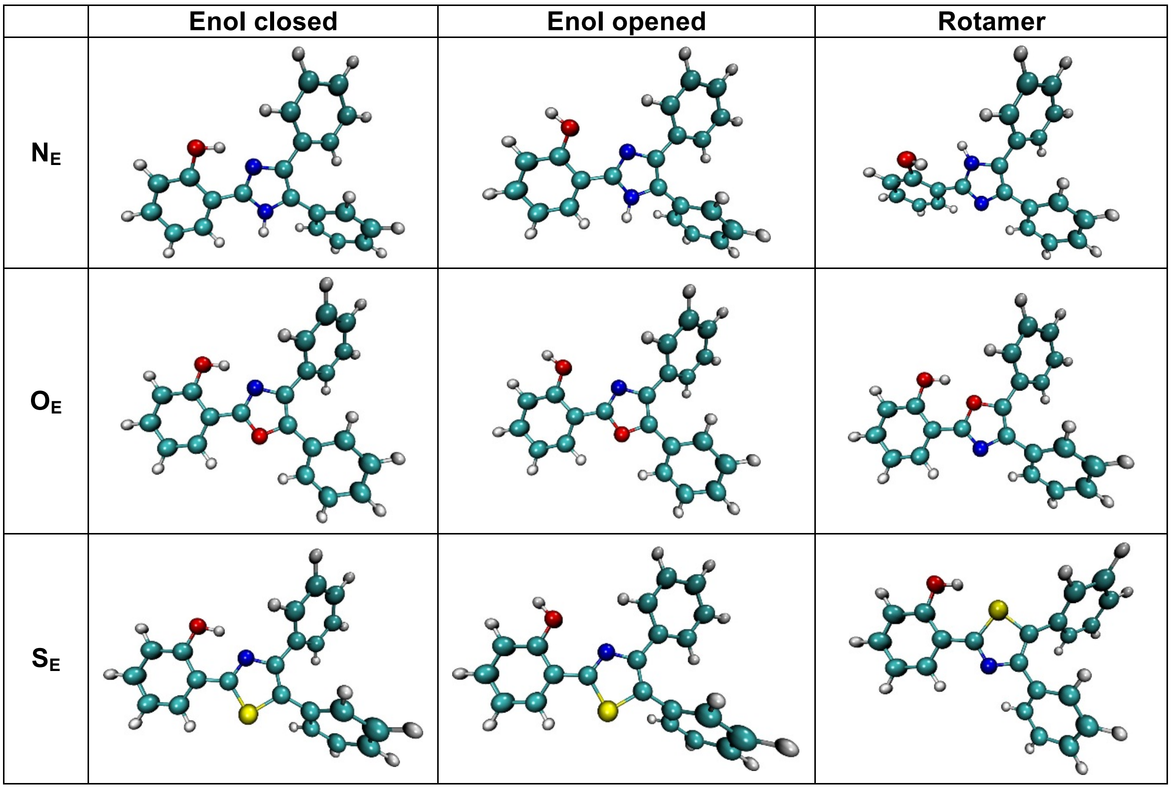

Figure S1: Optimized structure of N_E, O_E and S_E in enol closed forms, enol opened forms and their rotamers.

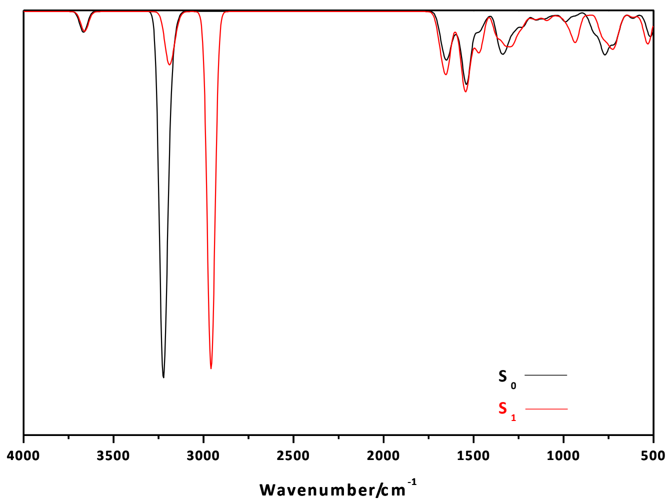

Figure S2: Vibrational spectrum of N_E in the ground and excited state in gas phase.

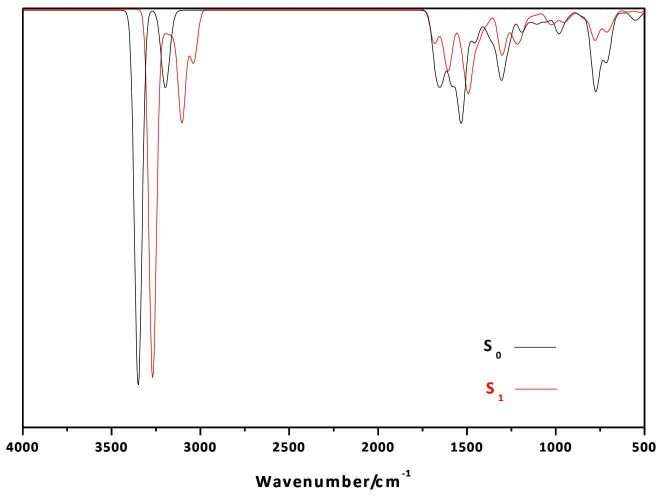

Figure S3: Vibrational spectrum of O_E in the ground and excited state in gas phase.

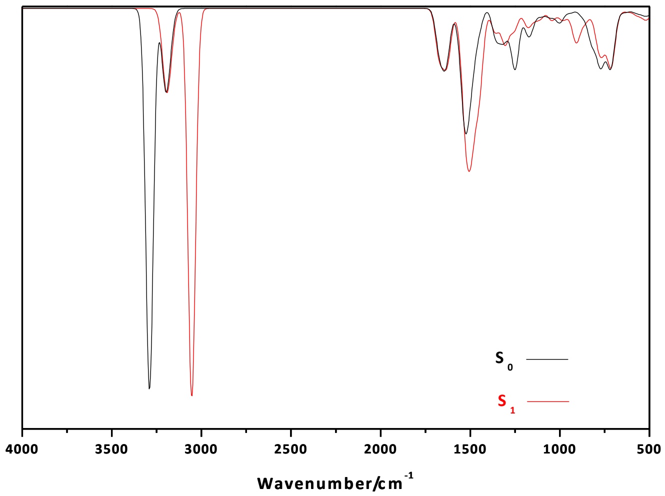

Figure S4: Vibrational spectrum of S_E in the ground and excited state in gas phase.

Table S2: HOMO → LUMO transition calculated with different functionals: theoretical absorption wavelength (λ_{abs}) and oscillator strength (f) calculated for N_E compound

Functional	% HF exchange	λ_{abs} (nm)	*f*
**B3LYP**	20	337.5	0.3850
**M06**	27	329.4	0.2948
**wB97X-D3**	22*	283.7	0.6434
**M06-2X**	54	274.6	0.4458

* Range separated hybrid functional with 22% short-range HF exchange, Grimme´s dispersion correction and range-separation parameter γ = 0.2

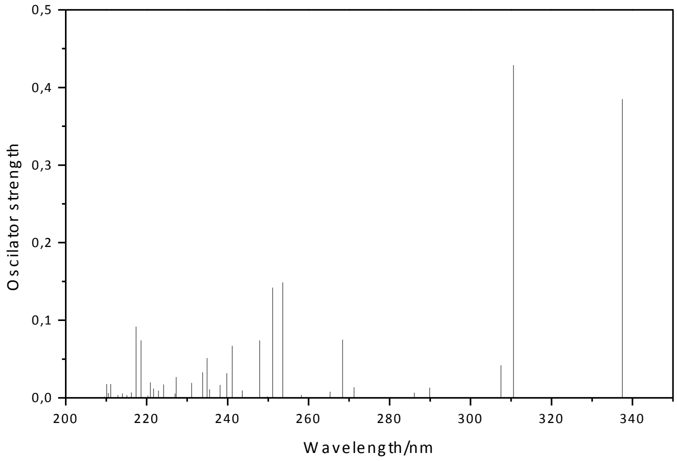

Figure S5: Absorption spectrum of N_E in gas phase.

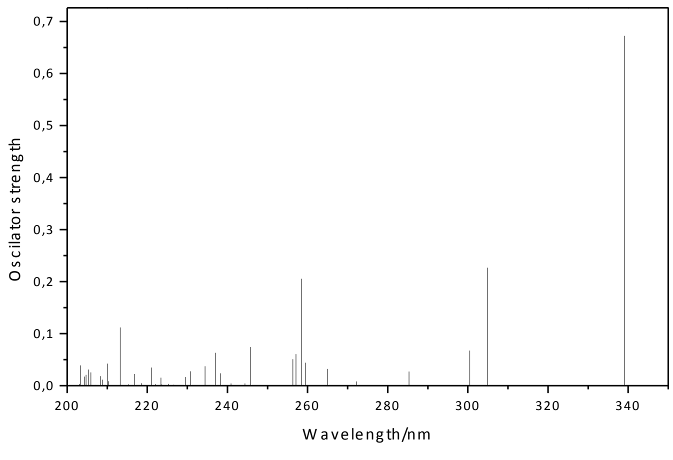

Figure S6: Absorption spectrum of O_E in gas phase.

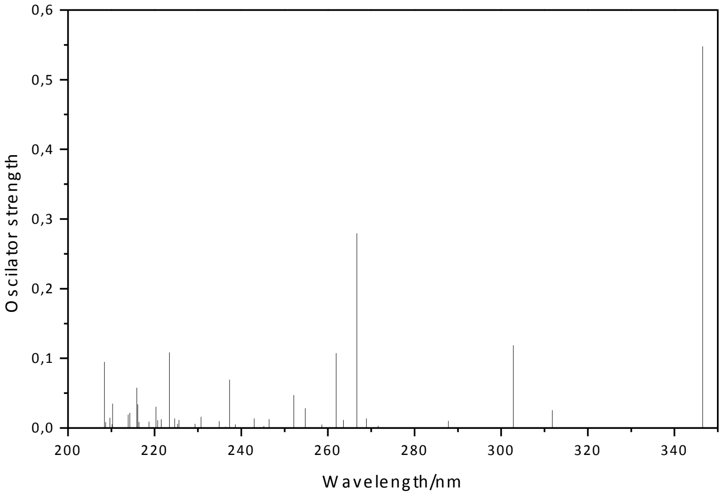

Figure S7: Absorption spectrum of S_E in gas phase.

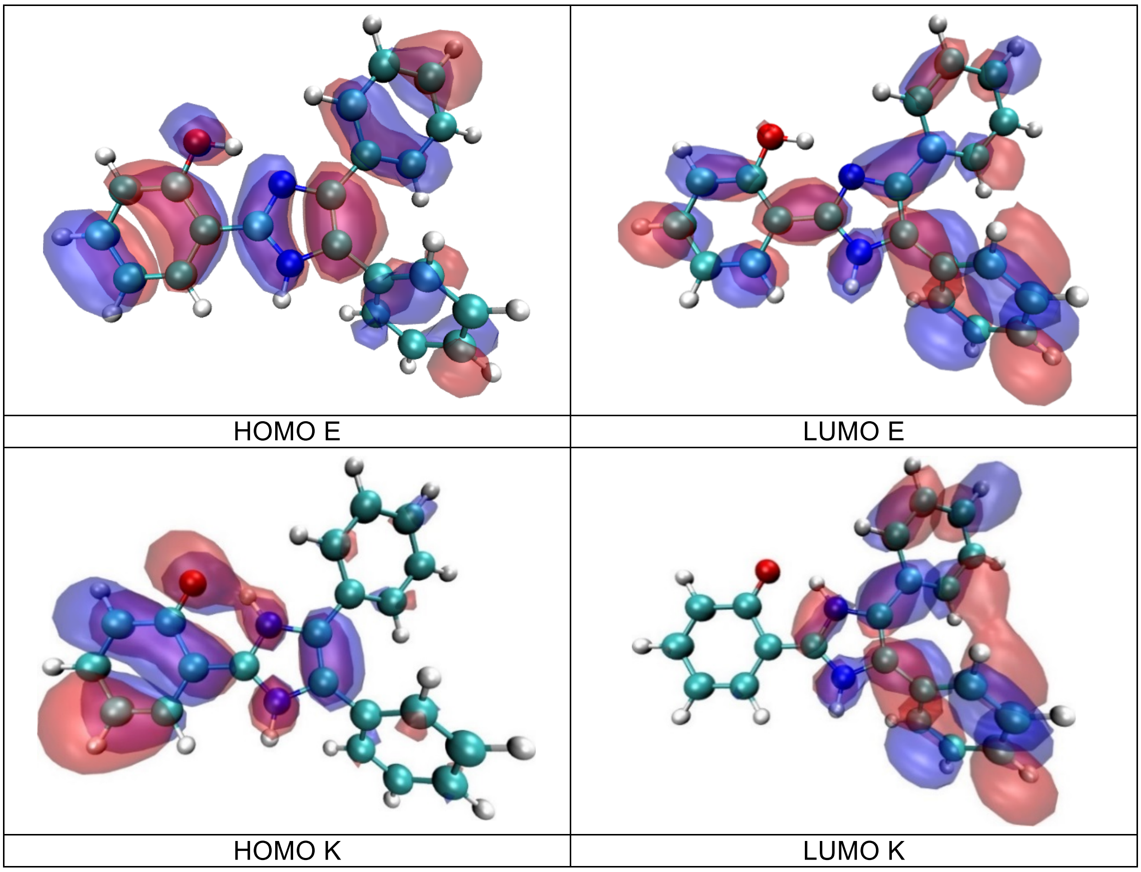

Figure S8: HOMO and LUMO orbitals of enolic (E) and ketonic (K) forms of triphenylimidazolic species.

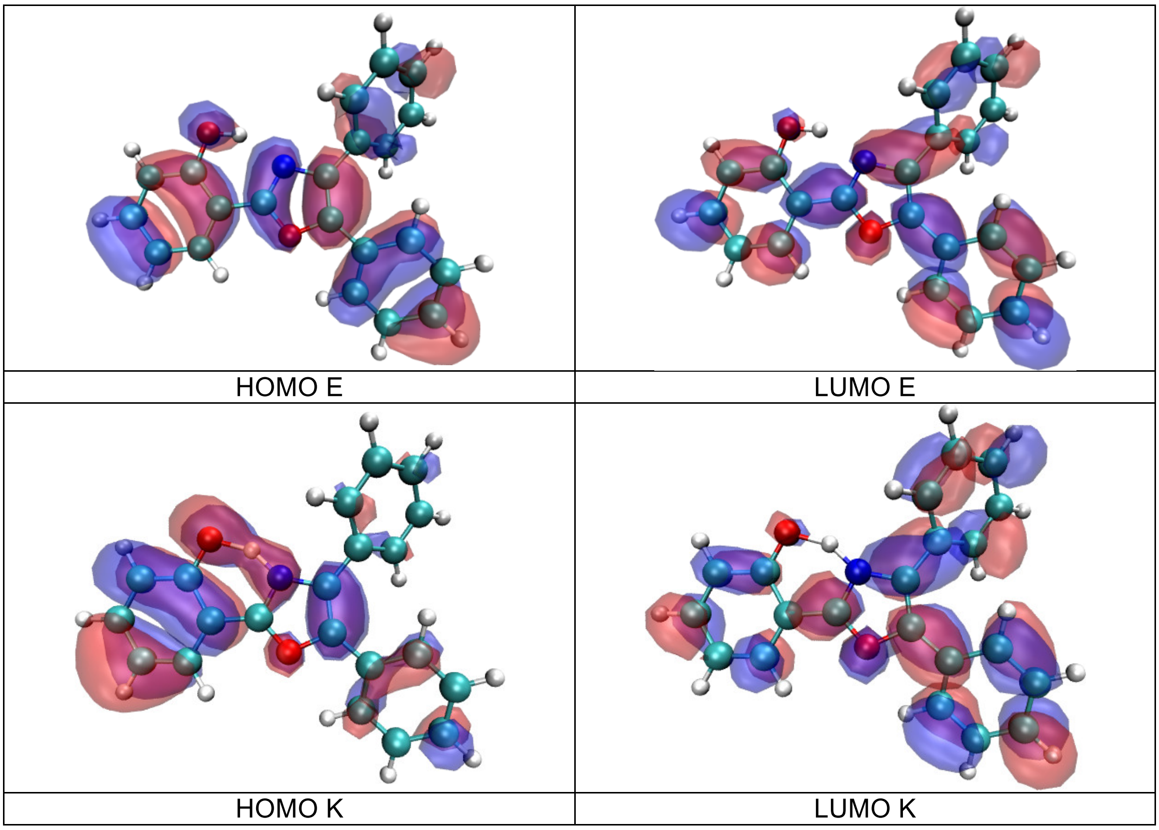

Figure S9: HOMO and LUMO orbitals of enolic (E) and ketonic (K) forms of triphenyloxazolic species.

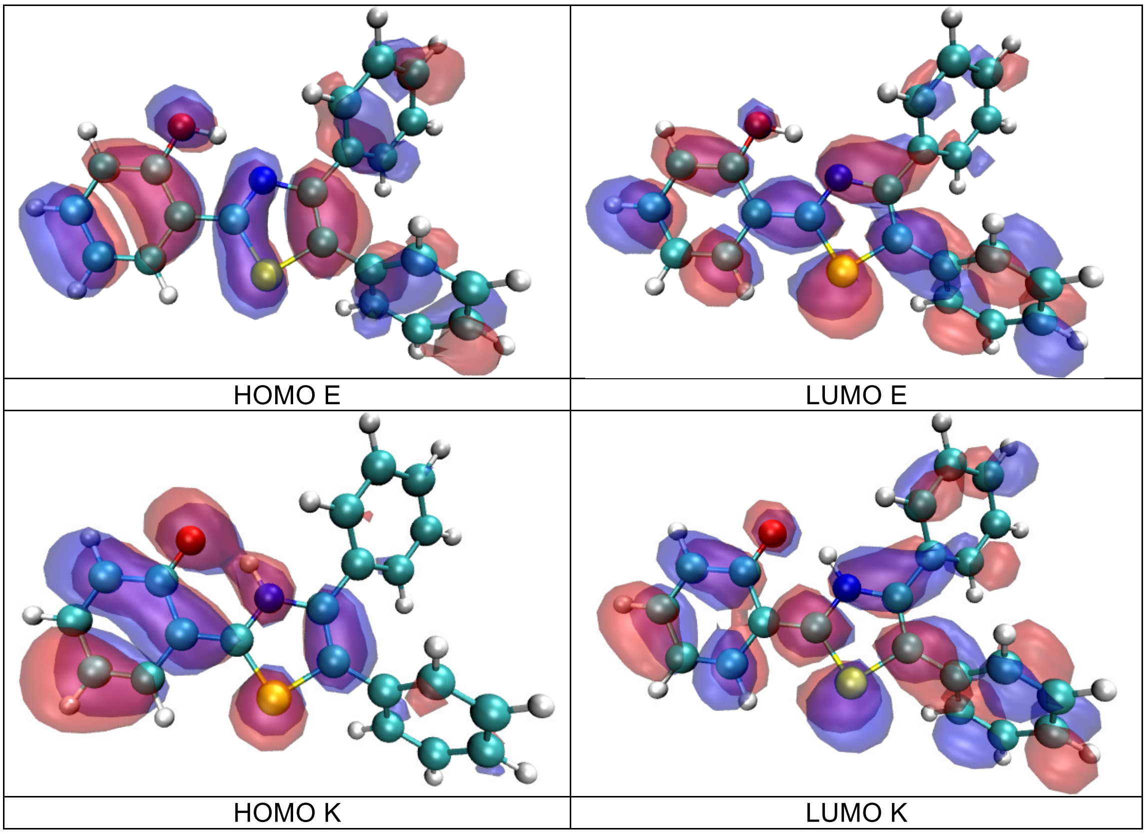

Figure S10: HOMO and LUMO orbitals of enolic (E) and ketonic (K) forms of triphenylthiazolic species.
